# Neutropenia exacerbates infection by *Acinetobacter baumannii* clinical isolates in a murine wound model

**DOI:** 10.3389/fmicb.2015.01134

**Published:** 2015-10-16

**Authors:** Laryssa M. Grguric-Smith, Hiu H. Lee, Jay A. Gandhi, Melissa B. Brennan, Carlos M. DeLeon-Rodriguez, Carolina Coelho, George Han, Luis R. Martinez

**Affiliations:** ^1^Department of Biomedical Sciences, Long Island University-PostBrookville, NY, USA; ^2^Department of Biomedical Sciences, College of Osteopathic Medicine, New York Institute of TechnologyOld Westbury, NY, USA; ^3^Department of Microbiology, Johns Hopkins School of Public HealthBaltimore, MD, USA; ^4^Centre for Molecular & Cellular Biology of Inflammation, Kings CollegeLondon, UK; ^5^Montefiore Medical Center, Division of Dermatology, Department of MedicineBronx, NY, USA

**Keywords:** *Acinetobacter baumannii*, collagen, cytokines, neutropenia, wound healing

## Abstract

The Gram negative coccobacillus *Acinetobacter baumannii* has become an increasingly prevalent cause of hospital-acquired infections in recent years. The majority of clinical *A. baumannii* isolates display high-level resistance to antimicrobials, which severely compromises our capacity to care for patients with *A. baumannii* disease. Neutrophils are of major importance in the host defense against microbial infections. However, the contribution of these cells of innate immunity in host resistance to cutaneous *A. baumannii* infection has not been directly investigated. Hence, we hypothesized that depletion of neutrophils increases severity of bacterial disease in an experimental *A. baumannii* murine wound model. In this study, the Ly-6G-specific monoclonal antibody (mAb), 1A8, was used to generate neutropenic mice and the pathogenesis of several *A. baumannii* clinical isolates on wounded cutaneous tissue was investigated. We demonstrated that neutrophil depletion enhances bacterial burden using colony forming unit determinations. Also, mAb 1A8 reduces global measurements of wound healing in *A. baumannii*-infected animals. Interestingly, histological analysis of cutaneous tissue excised from *A. baumannii*-infected animals treated with mAb 1A8 displays enhanced collagen deposition. Furthermore, neutropenia and *A. baumannii* infection alter pro-inflammatory cytokine release leading to severe microbial disease. Our findings provide a better understanding of the impact of these innate immune cells in controlling *A. baumannii* skin infections.

## Introduction

The Gram-negative coccobacillus *Acinetobacter baumannii* has become an increasingly prevalent cause of hospital-acquired infections during the last 15 years (Howard et al., 2012). This pathogen is a frequent cause of pneumonia and has been identified as the etiologic agent of complicated infections, especially wound infections (Johnson et al., 2007). For instance, the organism causes 2.1% of intensive care units-acquired skin/soft tissue infections (Gaynes et al., 2005) and was isolated from >30% of combat victims with open tibial fractures in the Middle East (Johnson et al., 2007). Moreover, the majority of clinical *A. baumannii* isolates display high-level resistance to antimicrobials, which severely compromises our capacity to care for patients with *A. baumannii* disease (Mihu and Martinez, 2011; Howard et al., 2012). Despite its clinical importance, little is known about the cellular and molecular mechanisms of host defense against cutaneous *A. baumannii* infection.

Neutrophils play an important role in early control of acute bacterial infections by killing bacteria through powerful oxidative and non-oxidative mechanisms and the production of pro-inflammatory cytokines (Mantovani et al., 2011). Clinical studies have shown that *A. baumannii* is one of the most frequently isolated gram-negative bacteria in neutropenic febrile patients in nosocomial settings, (Karim et al., 1991; Fukuta et al., 2013; Yadegarynia et al., 2013; Kim et al., 2014) particularly after prolonged hospitalization (Wisplinghoff et al., 2004). Previous studies have also shown that neutrophils (van Faassen et al., 2007; Qiu et al., 2009) and neutrophil-recruiting chemokines (Zhao et al., 2011) are present at the site of *A. baumannii* infection, and neutrophil granule extract is bactericidal to other species of *Acinetobacter* (Loeffelholz and Modrzakowski, 1988). However, the contribution of neutrophils in host resistance to cutaneous *A. baumannii* infection has not been directly investigated.

Most of our current knowledge about neutrophil function in the setting of *A. baumannii* infection originates from mice treated with cyclophosphamide, (Qiu et al., 2009; Lin et al., 2012; Manepalli et al., 2013; Thompson et al., 2014; Bruhn et al., 2015) a cytotoxic alkylating agent widely used for the treatment of neoplastic and severe autoimmune diseases. Cyclophosphamide suppresses myelopoiesis resulting in neutrophil depletion in murine models (Zuluaga et al., 2006). Moreover, cyclophosphamide inhibits a suppressor response that normally prevents activation of effector T cells (Yasunami and Bach, 1988). The exacerbation of inflammatory responses and blockade of suppressive activity after cyclophosphamide treatment is consistent with the suggestion that this agent preferentially depletes suppressor or regulatory T cells (Yasunami and Bach, 1988; Ghiringhelli et al., 2004). Additionally, cyclophosphamide reduces the number of peripheral and circulating macrophages, (Santosuosso et al., 2002) phagocytic cells that are capable of detecting and eliminating *A. baumannii* as well as initiating a host early immune response (Qiu et al., 2012). Nevertheless, while cyclophosphamide is useful to study immunosuppression in rodents challenged with *A. baumannii*, it is not necessarily an ideal model to study specific neutrophil function.

Depletion of neutrophils with antibodies to Ly-6G (Breslow et al., 2011) and Gr-1 (van Faassen et al., 2007) have shown that *A. baumannii* establishes infections in a murine model of pneumonia. Here, the Ly-6G-specific monoclonal antibody (mAb), 1A8, has been used to deplete neutrophils in mice and investigate the role of these cells in host defense (Dovi et al., 2003). We hypothesized that depletion of neutrophils would increase severity of *A. baumannii* disease in an experimental murine wound model. We showed that neutrophil depletion increases bacterial load in cutaneous tissue and alters the host immune response using distinct *A. baumannii* clinical isolates. Our findings provide a deeper understanding of the impact of neutrophils in controlling *A. baumannii* skin infections which may lead to the development of more effective therapeutic strategies.

## Materials and Methods

### Acinetobacter baumannii

A total of 7 *A. baumannii* clinical isolates (0057, 1422, 1611, 2098, 2231, 3559, and 7405) were included in the study. They were isolated from blood and wound cultures at the Walter Reed Medical Center, Washington, DC, USA and Montefiore Medical Center, Bronx, NY, USA. The antimicrobial susceptibility profile for each clinical isolate tested in this study was previously published (Orsinger-Jacobsen et al., 2013). The strains were stored at –80°C in brain heart infusion (BHI; Becton Dickinson (BD) Biosciences, Franklin Lakes, NJ, USA) broth with 40% glycerol until use. Test organisms were grown in a Tryptic Soy broth (TSB; MP Biomedicals, LLC, Solon, OH, USA) overnight at 37°C using a rotary shaker set at 150 rpm. Growth was monitored by measuring the optical density at 600 nm using a microtiter plate reader (OD_600_; Bio-Tek, Winooski, VT, USA).

### MAb 1A8 Administration

Female Balb/c mice (6–8 weeks; National Cancer Institute, Frederick, MD, USA) were injected intraperitoneally (i.p.) with a single dose of 500 μg/mL of mAb 1A8 (Rat anti-mouse IgG2a; (BD) in a 100 μL of sterile PBS. Control animals were injected with irrelevant IgG2a antibody (control IgG2a; Southern Biotech, Birmingham, AL, USA). Three days after mAb administration, neutrophil depletion was confirmed by differential leukocyte count in all experimental animals using a Hema 3 Stat Pack (Fisher HealthCare, Kalamazoo, MI, USA) and light microscopy.

### Flow-Cytometry

For flow cytometry staining, primary cells were isolated from blood withdrawn from five mice treated with mAb 1A8 or irrelevant antibody as described above; the cells were washed and then stained with fluorescence-labeled antibodies. Anti-Ly-6G-FITC (neutrophils) and its isotype control were purchased from (BD). Samples were processed on a LSRII flow cytometer (BD) and were analyzed using FlowJo software.

### *In Vivo* Wound Model and *A. baumannii* Infection

At day 3 after treatment, 1A8- and control IgG2a-treated mice were anesthetized with 100 mg/kg ketamine (Keta-set^®^, Fort Dodge, IA, USA) and 10 mg/kg xylazine (Anased^®^, Shenandoah, IA, USA), the hair on their backs removed, and the skin disinfected with iodine. Then, single punch biopsies were performed, resulting in 5-mm diameter full-thickness excision wounds. Thereafter, a suspension containing 10^7^ *A. baumannii* colony-forming units (CFU) in PBS was inoculated directly onto the wound of 1A8- and control IgG2a-treated mice. 1A8- and PBS-treated but uninfected mice were used as additional controls. Photographs of the wounds were taken on days 3 and 7 to grossly document wound healing, utilizing a ruler for determining scale. Additionally, the dimensions of each wound were measured every other day using a dial caliper and were performed by two different operators in a blinded fashion for each mouse. Eight animals per group were euthanized at days 3 and 7 after infection and wound tissues were excised for processing for histology, CFU determinations and gene expression.

### Ethics Statement

All animal studies were conducted according to the experimental practices and standards approved by the Institutional Animal Care and Use Committee (IACUC) at Long Island University (Protocol #: 11-3). The IACUC at Long Island University approved this study.

### CFU Determinations in Tissues

At days 3 and 7 post-infection, mouse cutaneous tissues were excised and homogenized in sterile PBS. Serial dilutions of homogenates were performed; a 100 μL suspension of each sample was then plated on Tryptic Soy Agar (TSA; MP Biomedicals, LLC) plates and incubated at 37°C for 24 h. Quantification of viable bacterial cells was determined by CFU counts and the results were normalized by tissue weights.

### Histological Processing

At days 3 and 7 post-infection, wounded tissues were excised from euthanized mice; the tissues were fixed in 10% formalin and embedded in paraffin. Four micrometer vertical sections were cut and then fixed to glass slides and subjected to Haematoxylin and Eosin (H&E), Gram, MPO, or collagen type I (Santa Cruz Biotechnology, Dallas, TX, USA) mAb staining to assess morphology, bacterial burden, neutrophil infiltration, or collagen deposition, respectively. The slides were visualized using an Axiovert 40CFL inverted microscope (Carl Zeiss, Thornwood, NY, USA), and images were captured with an AxioCam MrC digital camera using the Zen 2011 digital imaging software. Quantification of the collagen staining was carried out using ImageJ software using threshold filters to isolate the stain and measurement of colorimetric intensity.

### Real-time PCR for *COL1* and *COL3* Gene Expression in Wounded Tissue

*COL1* encodes for collagen type I which is present in scar tissue, the end product when tissue heals by repair. *COL3* encodes for collagen type III, found in extensible connective tissues such as skin, lung, and the vascular system, frequently in association with type I collagen. Briefly, seven day post-infection excised tissues were subjected to homogenization, cells were collected and washed, and then RNA was isolated using an RNeasy kit (QIAGEN, Valencia, CA, USA). *COL1* and *COL3* expression were analyzed by quantitative reverse transcription-PCR (qRT-PCR) as previously described (Han et al., 2012).

### Cytokine, Myeloperoxidase, and Superoxide Determinations

Three mice per group were sacrificed 3 and 7 days post-infection. Wounded tissues were excised and homogenized in PBS with protease inhibitors (Complete Mini; Roche, Ridgefield, CT, USA). Cell debris was removed from homogenates by centrifugation at 6,000 *g* for 10 min. Samples were stored at –80°C until tested.

#### (i) Cytokines

Supernatants were tested for IFN-γ, TNF-α, IL-1β, and IL-6 by ELISA (BD). The limits of detection were 31.3 pg/mL for IFN-γ and 15.6 pg/mL for TNF-α, IL-1β, and IL-6.

#### (ii) Myeloperoxidase

Supernatants were tested for myeloperoxidase (MPO) by ELISA (Hycult Biotechnology, The Netherlands). MPO is an enzyme most abundantly produced by neutrophils respiratory burst. The limits of detection were 1 ng/mL for MPO.

#### (iii) Superoxide

Superoxide (O_2_–) produced in murine tissue supernatant was quantified after exposure to *A. baumannii* using a superoxide dismutase assay kit (EMD Millipore, Billerica, MA, USA).

### Statistical Analysis

Data were analyzed using Prism (GraphPad, LaJolla, CA, USA). Differences in neutrophil counts, MPO and superoxide levels, CFUs, and cytokine data were analyzed by the student’s *t*-test. Analyses of wound healing, collagen deposition, and gene expression data were done using analysis of variance (ANOVA) and adjusted by use of the Bonferroni correction. *P*-values of <0.05 were considered significant.

## Results

### MAb 1A8 Decreased Neutrophils in Blood and Skin Tissue of Treated Balb/c Mice

**FIGURE 1:**
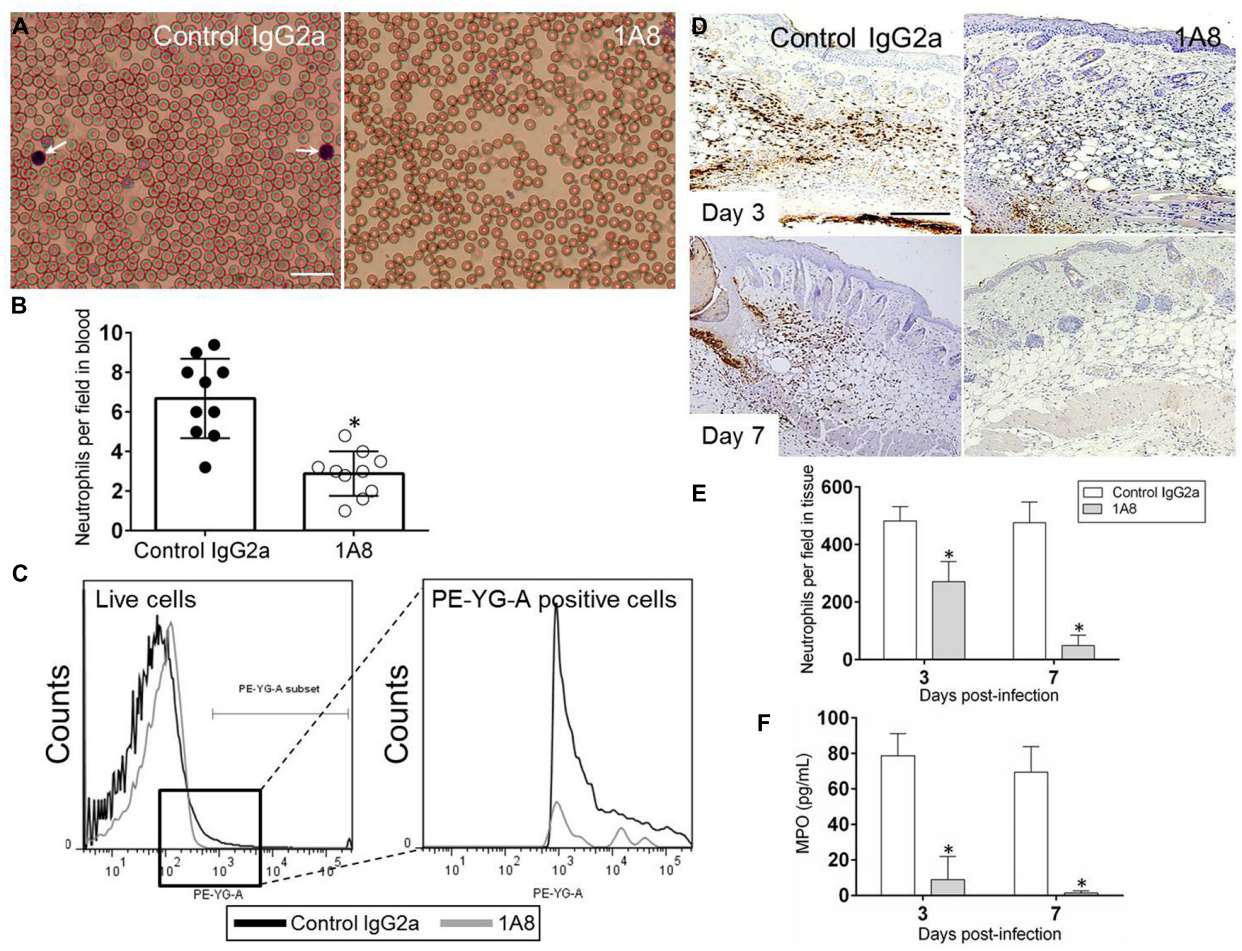
**MAb 1A8-treated animals display a low number of blood and skin tissue neutrophils. (A)** Light microscopy images of blood smears from control (isotype-matching IgG2a) or mAb 1A8-treated (1A8) mice pre-infection. Pictures were taken 3 days post-injection. Arrows indicate neutrophils. Scale bar: 20 μm. **(B)** Number of neutrophils per field in blood of irrelevant (control IgG2a) or 1A8-treated mice quantified 3 days post-injection. Each black or white circle represents the numbers of neutrophils per individual field. Bars and error bars denote average of ten counts and standard deviations, respectively. Asterisks denote *P*-value significance (^∗^*P* < 0.05) calculated using student’s *t*-test analysis. The experiment was performed thrice with similar results obtained. **(C)** The expression levels of the Ly-6G^+^ cells were analyzed by flow cytometry 3 days post-injection and a representative graph is shown. Primary cells were isolated from blood of animals (*n* = 5) injected with irrelevant or mAb 1A8. The experiments were performed twice with similar results obtained. **(D)** Immunohistochemistry (IHC) of myeloperoxidase (MPO) released by neutrophils in wounds removed from control IgG2a and 1A8-treated mice. MPO-specific monoclonal antibody (mAb) was used to stain MPO (brown) released in skin tissue indicative of neutrophil infiltration. Representative MPO-immunostained sections of the skin lesions are shown. Scale bars: 20 μm. **(E)** Number of neutrophils per field in wounded skin tissue of control and 1A8 animals. Data are given as the average number of neutrophils in 10 different fields, and error bars denote standard deviations. **(F)** MPO concentration in the supernatant of tissue homogenates excised from control and mAb 1A8-treated mice (*n* = 5 per group). Bars represent the mean values; error bars denote standard deviations. For **(E,F)**, asterisks denote *P*-value significance (^∗^*P* < 0.001) calculated using student’s *t*-test analysis. The experiments were performed twice with similar results obtained.

We examined whether mAb 1A8 administration depleted neutrophils in blood smears of Balb/c mice using differential leukocyte staining. Light microscopy images show apparent reduced numbers of neutrophils in blood of 1A8-treated mice, compared with IgG2a control mice (**Figure 1A**). Cell count analysis showed that 1A8-treated animals had significantly lower blood circulating neutrophils when compared to controls (*P* = 0.0224; **Figure 1B**). Flow cytometry analysis confirmed a significant decrease of Ly6-G^+^ cells in the blood of 1A8-injected mice (**Figure 1C**). Similarly, we determined whether mAb 1A8 injection reduced neutrophil infiltration in wounded tissue by immunohistochemistry (IHC; **Figures 1D,E**) and MPO analyses (**Figure 1F**). Both analyses showed that mAb 1A8-treated animals had significantly decreased neutrophil infiltration into the skin tissue compared to IgG2a controls (**Figure 1E**, Day 3 *P* = 0.0008, Day 7 *P* = 0.0001; **Figure 1F**, Day 3 *P* = 0.0001, Day 7 *P* = 0.0001).

### *Acinetobacter baumannii* Infected Mice Show Reduced Wound Healing Rate *In Vivo*

**FIGURE 2:**
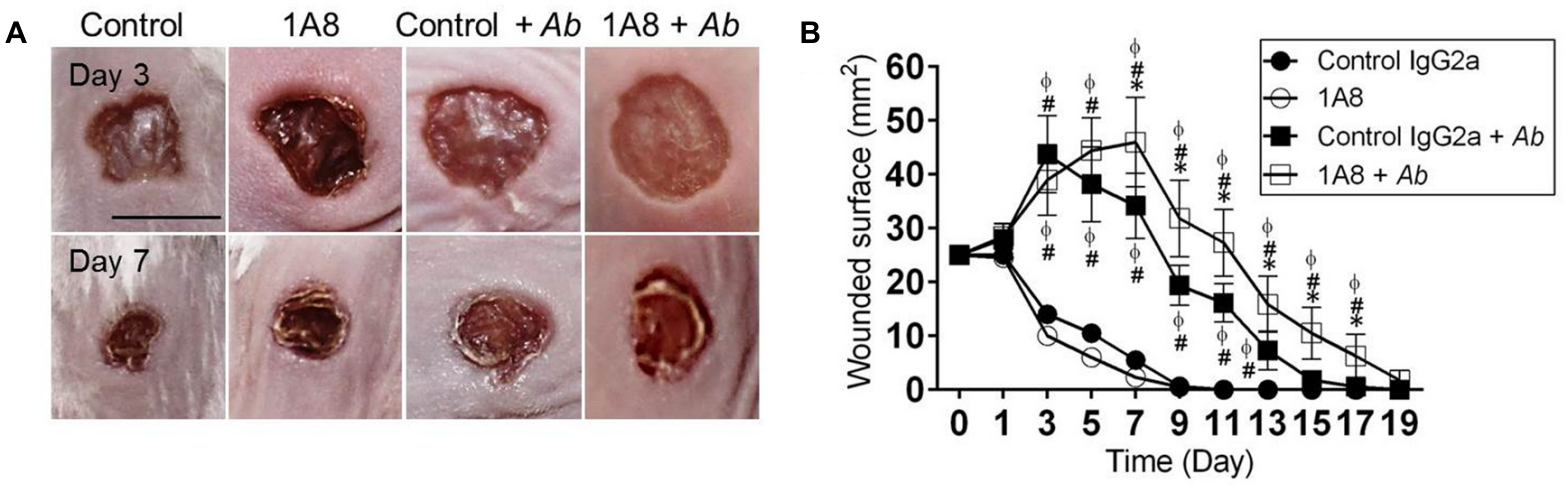
**Neutropenic mice with *Acinetobacter baumannii* (*Ab*) wound infection have reduced wound healing rate. (A)** Wounds of Balb/c mice isotype-matching mAb-treated (control IgG2a), mAb 1A8-treated (1A8), control IgG2a-treated *A. baumannii*-infected (control IgG2a + *A. baumannii*), and mAb 1A8-treated *A. baumannii*-infected (1A8 + *A. baumannii*), 3 and 7 days post-wounding and infection. Scale bar: 5 mm. **(B)** Wounded surface analysis of Balb/c mice skin lesions. For control and 1A8 groups, time points are the averages of the results for five measurements, and error bars denote standard deviations. For control IgG2a + *A. baumannii* and 1A8 + *A. baumannii* groups, time points are the averages of results for seven clinical isolates (*n* = 7) and error bars denote standard deviations. Symbols (∗, #, ϕ relative to control IgG2a + *A. baumannii*, 1A8, and control IgG2a, respectively) denote *P*-value significance (*P* < 0.01) calculated by analysis of variance (ANOVA). This experiment was performed twice with similar results obtained.

The effect of neutrophil depletion on *A. baumannii* infection and wound healing was investigated (**Figure 2**). Uninfected animals showed faster wound healing rates than *A. baumannii* infected groups (**Figures 2A,B**). At day 3, the eschars in control IgG2a and 1A8 wounds were ∼13.2 and 11.5 mm^2^ in surface, respectively, whereas eschars of control IgG2a + *A. baumannii* and 1A8 + *A. baumannii* wounds were ∼43.7 (compared to IgG2a *P* = 0.0001 and 1A8 *P* = 0.0001) and 38.9 mm^2^ (compared to IgG2a *P* = 0.0001 and 1A8 *P* = 0.0001; **Figure 2B**). At day 7, eschars in the control IgG2a and 1A8 groups were ∼5.3 and 2.5 mm^2^, respectively, whereas the eschars of control IgG2a and 1A8 *A. baumannii*-infected wounds were ∼34.2 (compared to IgG2a *P* = 0.0001 and 1A8 *P* = 0.0001) and 45.9 mm^2^ (compared to IgG2a *P* = 0.0001, 1A8 *P* = 0.0001, IgG2a + *A. baumannii P* = 0.0101), respectively (**Figure 2B**). At day 11, the wounds of control IgG2a and 1A8 groups reached complete closure, whereas eschars of control IgG2a + *A. baumannii* and 1A8 + *A. baumannii* wounds were ∼16.1 (compared to IgG2a, *P* = 0.0001 and 1A8, *P* = 0.0001) and 27.3 mm^2^ (compared to IgG2a *P* = 0.0001, 1A8 *P* = 0.0001, IgG2a + *A. baumannii P* = 0.0013), respectively. At day 15, the lesions of 1A8 + *A. baumannii* wounds were ∼10.5 mm^2^. On average, complete wound healing did not occur in the infected groups until day 15 (control IgG2a) and 19 (1A8; **Figure 2B**).

### 1A8-Treated Animals Displayed Increased Collagen Production in Cutaneous Tissue

**FIGURE 3:**
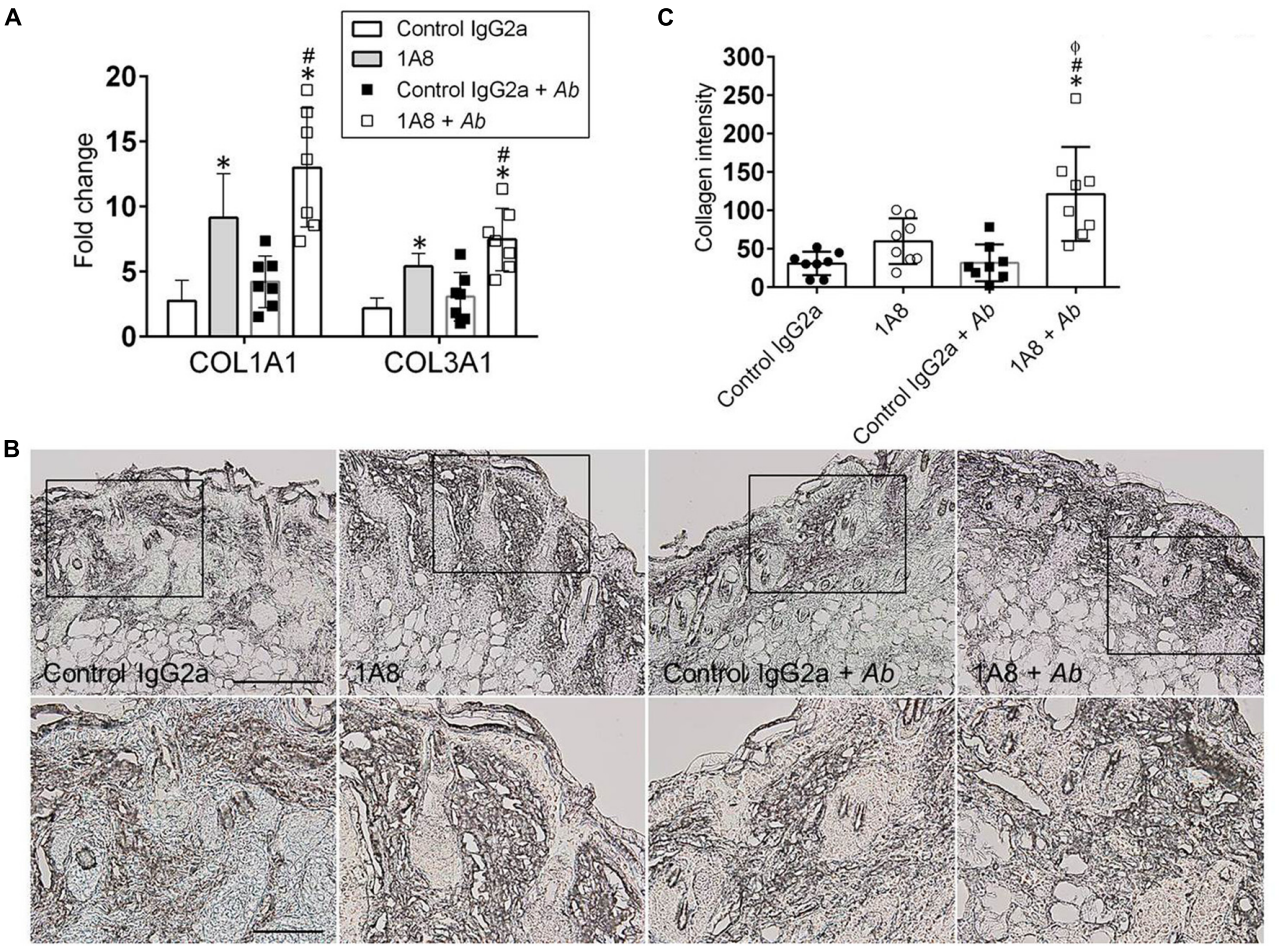
**Neutropenia promotes collagen deposition in cutaneous lesions of rodents. (A)** Gene expression analysis of collagen type I (COL1A) and III (COL3A) in murine dermal tissue. For control and 1A8 uninfected animals, bars are the averages of the results for five wounds, and error bars denote standard deviations. For control IgG2a- and 1A8-infected with *A. baumannii* groups, bars are the averages of results for seven clinical isolates (each symbol represent a strain; *n* = 7) and error bars denote standard deviations. ∗ and # indicate higher fold changes than control and *A. baumannii* groups, respectively. **(B)** IHC of collagen type I produced in wounds of isotype-matching mAb-treated (control IgG2a), mAb 1A8-treated (1A8), control IgG2a-treated *A. baumannii*-infected (control IgG2a + *A. baumannii*), and mAb 1A8-treated *A. baumannii*-infected (1A8 + *A. baumannii*) Balb/c mice, 7 days post-infection. Representative 20X (upper panel) and 40X (lower panel; magnified black boxes in upper panel) collagen type I-immunostained sections of the skin lesions are shown. The dark staining indicates collagen. Scale bar: 20 μm. **(C)** Quantitative measurement of collagen type I intensity in eight representative fields (each symbol) of the same size for control IgG2a, 1A8, control IgG2a + *A. baumannii*, and 1A8 + *A. baumannii* wounds. Data are given as the average of the results, and error bars denote standard deviations. ∗, #, ϕ indicates higher levels than control IgG2a, 1A8, *A. baumannii* groups, respectively. For **(A,C)**, *P*-value significance (*P* < 0.05) was calculated by ANOVA. Each experiment was performed twice and similar results were obtained.

The role of neutrophils on wound healing was further explored by examining whether neutropenia modify collagen deposition in wounded tissue. Gene expression of collagen types I and III was significantly increased in 1A8-treated animals as compared to control IgG2a (collagen I, *P* = 0.0013; collagen III, *P* = 0.0379), and in 1A8-treated and *A. baumannii*-infected animals as compared to control IgG2a and *A. baumannii*-infected (collagen I, *P* = 0.0001; collagen III, *P* = 0.0011) groups (**Figure 3A**). Moreover, IHC of collagen type I revealed denser collagen deposition and thicker collagen bundles within the epidermis and dermis of 1A8-treated wounds as compared to control IgG2a wounds, both in infected and non-infected models (**Figure 3B**). Furthermore, this was well-correlated with quantitative analysis of staining intensity (**Figure 3C**). Besides uninfected models, the 1A8-treated samples showed a consistent trend (1A8) and a significant increase (1A8 + *A. baumannii*) in collagen staining intensity, signifying augmented collagen deposition (**Figure 3C**).

### MAb 1A8-Injected Mice Displayed a Higher Bacterial Burden in Cutaneous Lesions

**FIGURE 4:**
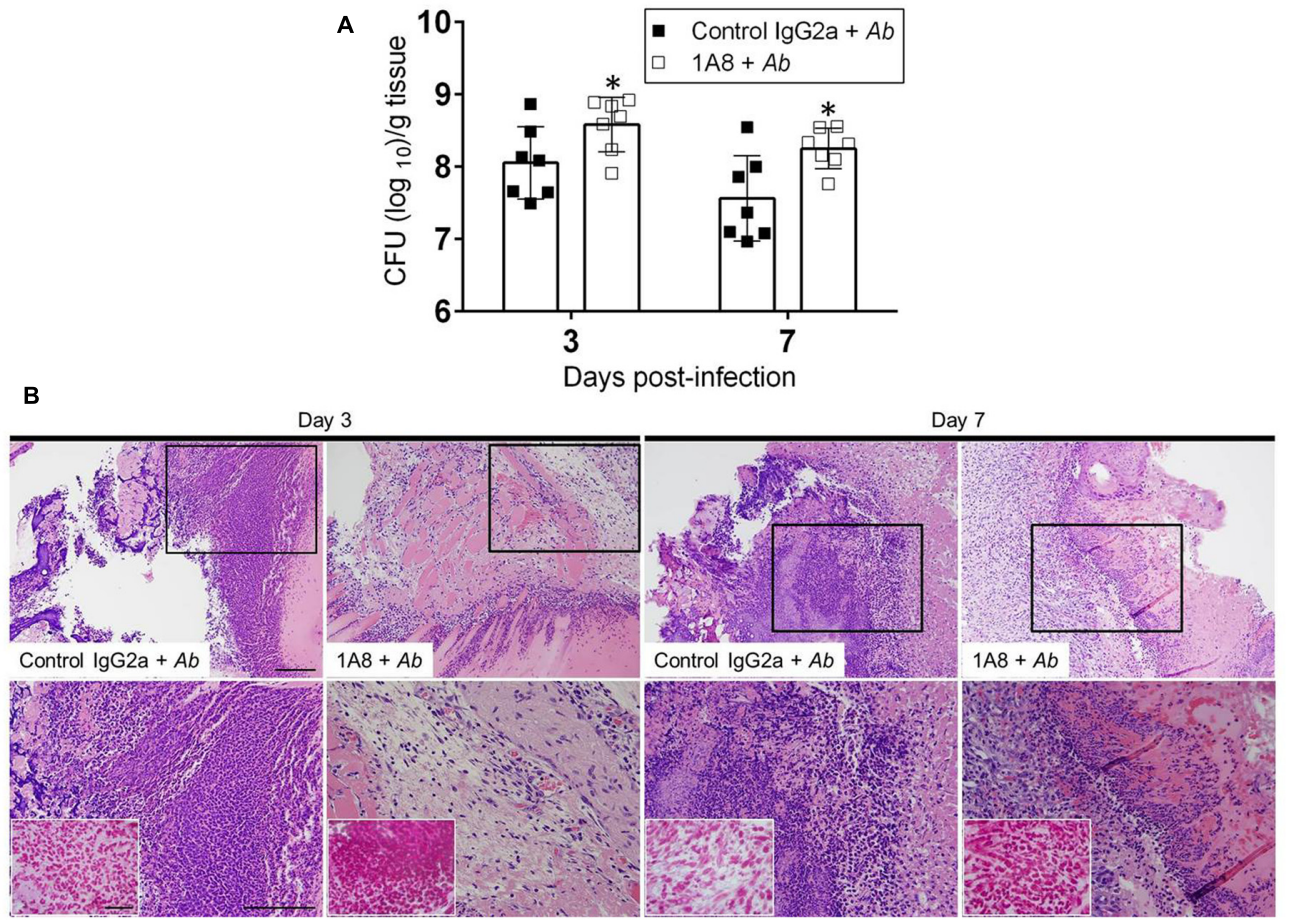
**Neutropenic mice show high bacterial burden in superficial skin lesions. (A)** Wound bacterial burden (CFU; colony forming units) in control IgG2a and mAb 1A8-treated mice infected with 10^7^ *A. baumannii* CFU. Three and seven days post-infection, infected skin tissue was removed from mice and bacterial burden determined. Bars are the averages of the results for seven clinical isolates (each symbol represent a strain; *n* = 7), and error bars denote standard deviations. Asterisks denote *P*-value significance (^∗^*P* < 0.05) calculated by student’s *t*-test analysis. This experiment was performed twice with similar results obtained. **(B)** Histological analysis of Balb/c mice infected with *A. baumannii* 0057, days 3 and 7. Representative 20X (upper panel) and 40X (lower panel; magnified black boxes in upper panel) H&E-stained sections of the skin lesions are shown with the *insets* representing Gram staining for *A. baumannii* cells (shown in pink-red spots; lower panel). Scale bars: 20 μm.

The role of neutrophils in killing *A. baumannii* in cutaneous lesions of mice was investigated. Three and seven days after infection, wounds were removed from control IgG2a or 1A8-treated and *A. baumannii* infected animals and plated on TSA. MAb 1A8-treated wounds evinced significantly higher microbial burden than did the control IgG2a wounds on days 3 (IgG2a 10^8.054^ CFU; 1A8 10^8.583^ CFU; *P* = 0.0363) and 7 (IgG2a 10^7.560^ CFU; 1A8 10^8.253^ CFU; *P* = 0.0298; **Figure 4A**). Histological examinations revealed that uninfected wounds quickly regained normal epidermal and dermal structure; both in control IgG2a and 1A8 treated mice (data not shown). However, inoculation of *A. baumannii* resulted in full-thickness wounds with an intense inflammatory infiltrate, persisting through both day 3 and day 7 after wounding in control IgG2a-treated mice (**Figure 4B**; upper panel 20X; lower panel 40X). Likewise, full-thickness wounds with prominent serum crust were observed in the *A. baumannii*-infected 1A8-treated wounds, but with significantly reduced inflammatory infiltrate. Gram stain reveals increased concentration of Gram-negative species within the *A. baumannii*-infected 1A8 treated wounds as compared to the *A. baumannii*-infected IgG2a wounds (**Figure 4B**; insets; lower panel).

### MAb 1A8 Treatment Reduces Neutrophil Infiltration to Wounded Tissues

**FIGURE 5:**
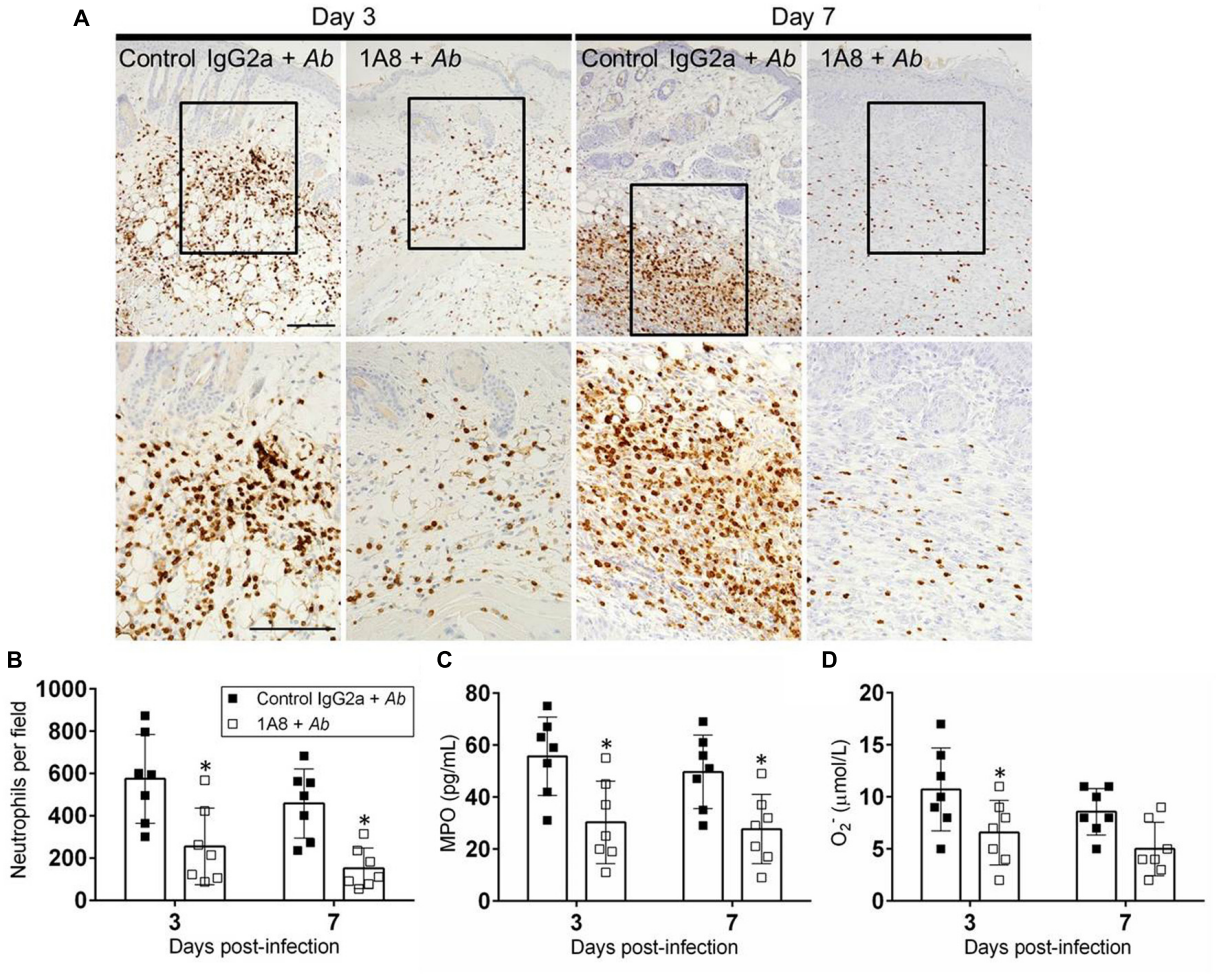
**MAb 1A8 administration decreases cutaneous neutrophil infiltration. (A)** IHC of MPO released by neutrophils in wounds removed from *A. baumannii*-infected (control IgG2a + *A. baumannii*), and mAb 1A8-treated *A. baumannii*-infected (1A8 + *A. baumannii*) mice. MPO-specific mAb was used to stain MPO (dark) released in skin tissue indicative of neutrophil infiltration. *Inset* shows restricted dermal accumulation of neutrophils. Representative 20X (upper panel) and 40X (lower panel; magnified black boxes in upper panel) MPO-immunostained sections of the skin lesions are shown. Scale bars: 20 μm. **(B)** Number of neutrophils per field in wounded skin tissue of control IgG2a + *A. baumannii* and 1A8 + *A. baumannii* animals. **(C)** MPO concentration in the supernatant of tissue homogenates excised from control IgG2a- and mAb 1A8-treated *A. baumannii*-infected mice. **(D)** Superoxide (O_2_^-^) production was quantified by measurement of SOD1 activity in tissue homogenates excised from control IgG2a + *A. baumannii* and 1A8 + *A. baumannii* mice. For **(B–D)**, bars represent the mean values for seven clinical isolates (each symbol); error bars denote standard deviations. Asterisks denote *P*-value significance (^∗^*P* < 0.05) calculated using student’s *t*-test analysis. The experiments were performed twice with similar results obtained.

We investigated the effect of mAb 1A8 administration in neutrophil migration to the wounded area during *A. baumannii* infection (**Figure 5**). Neutrophil infiltration was evaluated by neutrophil counts and measuring the production of MPO in the cutaneous lesions. On day 3, staining was mostly confined to scattered areas of the dermal tissue with more neutrophil infiltration in control mAb IgG2a-treated *A. baumannii*-infected wounds compared to mAb 1A8-treated *A. baumannii*-infected wounds (**Figure 5A**). On day 7, IgG2a-treated *A. baumannii*-infected wounds showcased localized neutrophil recruitment to the dermal and hypodermal areas of the wounded tissue (**Figure 5A**). In contrast, mAb 1A8-treated and *A. baumannii*-infected wounds displayed a reduced distributed neutrophil infiltration. Neutrophil counts revealed that wounds of mAb 1A8-injected group displayed significantly lower numbers of neutrophils than did the control IgG2a-treated *A. baumannii*-infected group (Day 3 *P* = 0.0398, Day 7 *P* = 0.0305; **Figure 5B**). Additionally, our quantitative analysis confirmed the presence of lower levels of MPO in wounds of 1A8-treated-*A. baumannii* infected mice when compared to wounds of control IgG2a-treated *A. baumannii* infected mice (Day 3 *P* = 0.0003, Day 7 *P* = 0.0012; **Figure 5C**). Finally, we measured the levels of O_2_– produced in wounded skin tissue. On day 3, the wounds of mAb 1A8-injected and *A. baumannii*-infected group showed significantly reduced O_2_– levels when compared to control IgG2a-infected groups (*P* = 0.0027; **Figure 5D**).

### MAb 1A8 Administration Modifies Cytokine Levels in Cutaneous Lesions

**FIGURE 6:**
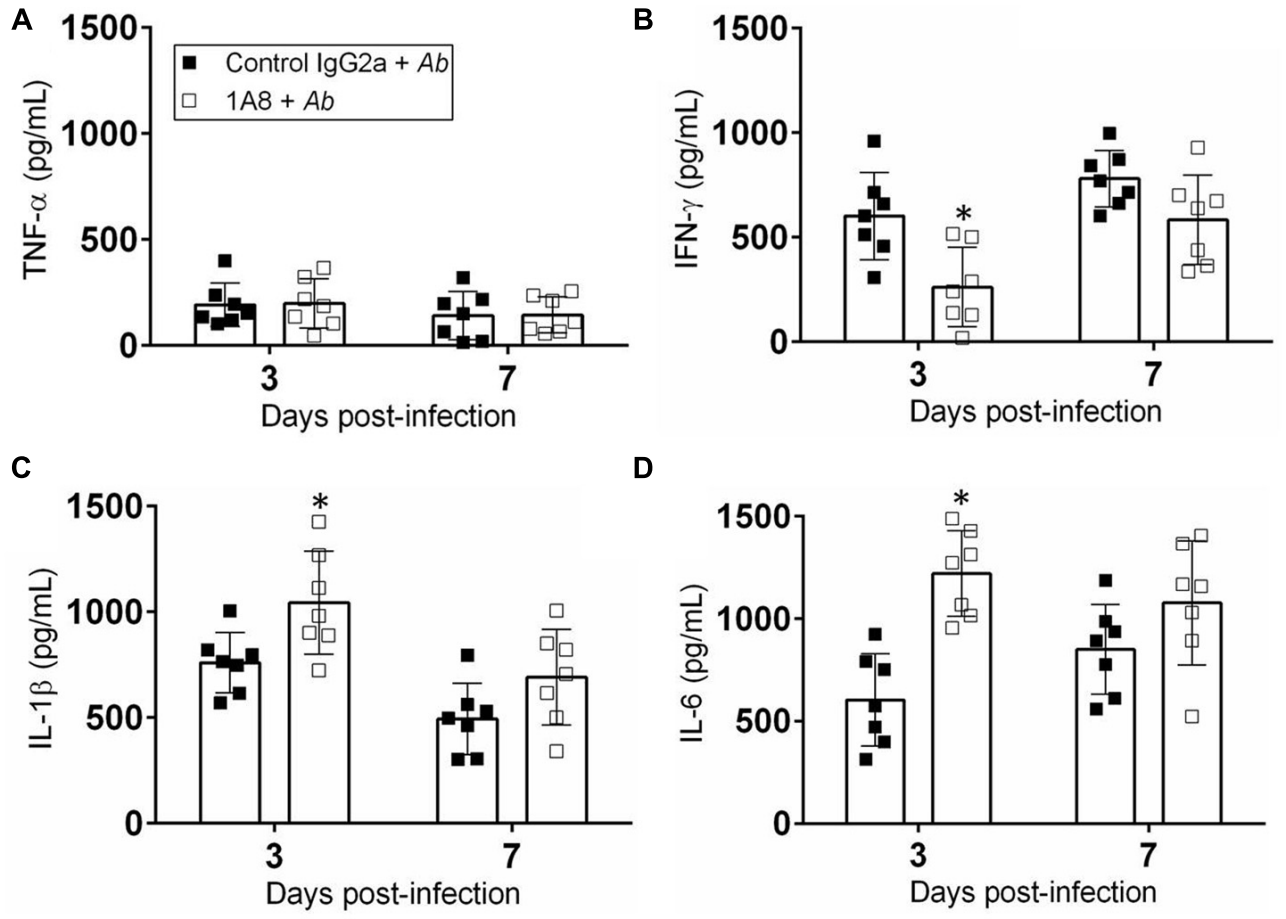
**Neutropenia altered pro-inflammatory cytokine production.** Homogenates of extracted wounded tissue from control IgG2a + *A. baumannii* and 1A8 + *A. baumannii* mice, 3 and 7 days post-infection were prepared and the supernatants were analyzed for **(A)** TNF-α, **(B)** IFN-γ, **(C)** IL-1β, and **(D)** IL-6 levels. Bars represent the mean values for seven clinical isolates (each symbol); error bars denote standard deviations. For **(A–D)**, asterisks denote *P*-value significance (^∗^*P* < 0.05) calculated using student’s *t*-test analysis. Cytokine quantification was performed thrice with similar results obtained.

We measured the cytokine response in the tissue homogenates of wounded mice incubated with *A. baumannii* after exposure to mAb 1A8 (**Figure 6**). Homogenates of wounded mice treated with either control IgG2a or 1A8 and infected with *A. baumannii* showed no differences in TNF-α production (**Figure 6A**). Wounded tissue of 1A8-treated-*A. baumannii*-infected animals contained significantly lower quantities of IFN-γ relative to the control IgG2a-treated-*A. baumannii*-infected groups 3 days post-wounding (*P* = 0.0078; **Figure 6B**). On day 3 post-wounding, IL-1β production was significantly increased in the 1A8-treated-*A. baumannii*-infected group when compared to the control group (*P* = 0.0204; **Figure 6C**). On day 7, the levels of IL-1β were decreased in both groups as compared to day 3. Similarly, there was a significant early increase in IL-6 production in 1A8-treated group compared to control IgG2a group (*P* = 0.0002; **Figure 6D**).

## Discussion

*Acinetobacter baumannii* is an opportunistic pathogen that causes hospital-related infections, especially pneumonia and sepsis, increasing morbidity and mortality (Karageorgopoulos et al., 2008). *A. baumannii* gained notoriety as a major causative agent of skin and soft tissue infections in soldiers injured in combat, surgical wounds, and ulcers (Sebeny et al., 2008). This bacterium has developed efficient mechanisms of drug resistance against the most commonly prescribed antimicrobials, making *A. baumannii* infections difficult to treat worldwide. Although progress has been made in understanding the causes and role of innate immunity in *A. baumannii*-related pneumonia, little is known about the cellular and molecular mechanisms of host defense in the setting of cutaneous infections. Since multi-drug resistant *A. baumannii* can colonize the skin, the frequency of cutaneous infections have increased in recent years, and neutrophils are an essential part of the initial immune response against bacterial infections and tissue damage, we used mAb 1A8 to deplete these cells of innate immunity in mice and explored their function on *A. baumannii* cutaneous infection.

Due to the variability in virulence between *A. baumannii* isolates described in earlier studies (Eveillard et al., 2010; Jacobs et al., 2014; Bruhn et al., 2015), we examined multiple strains regarding pathogenesis and wound healing in the cutaneous model. Although *A. baumannii* strain 1422, which has been described as a strong biofilm former (Orsinger-Jacobsen et al., 2013), was the only strain that showed hyper virulence correlating high CFU numbers and increased wound size (data not shown), on average, bacterial high density alone did not influence *A. baumannii* cutaneous damage in control IgG2a or 1A8-treated animals. These findings are in agreement with results presented by other investigators demonstrating that differences in virulence between *A. baumannii* strains are not only associated to bacterial proliferation (Eveillard et al., 2010). Moreover, the fact that neutrophil depletion allows the microbe to easily colonize wounded tissue highlights the importance of these cells of the innate immunity in controlling infections. Recently, Bruhn et al. (2015) reported that *A. baumannii* virulence showed by distinct strains may be determined by the ability of the organisms to evade innate immune effectors resulting in high microbial numbers established early after infection and progressing to septicemia.

Previously, Russo et al. (2008) described a rat soft-tissue model to screen *A. baumannii* virulence factors and antimicrobial reagents but the host response against this bacterium was not investigated. Additionally, an excisional, murine *A. baumannii* infection wound model comparable to our model was recently described and used to investigate *A. baumannii* biofilm formation and antibiotic efficacy (Thompson et al., 2014). In that model, cyclophosphamide is used as a neutropenic agent to establish an *A. baumannii* persistent infection. Yet, cyclophosphamide also preferentially depletes suppressor or regulatory T cells (Yasunami and Bach, 1988; Ghiringhelli et al., 2004) and affects circulating macrophages (Santosuosso et al., 2002). The advantage of using the mAb 1A8 over cyclophosphamide to deplete neutrophils is that it acts preferentially on these cells of innate immunity, making neutrophil function studies more reliable. Nevertheless, cyclophosphamide, while not specific for certain immune cells, is still a valuable model in understanding *A. baumannii* pathogenesis (Lin et al., 2012; Manepalli et al., 2013; Thompson et al., 2014).

Many studies used the anti-Gr-1 mAb RB6-8C5 as a neutrophil-depleting agent. For example, RB6-8C5-induced neutropenia in C57BL/6 and Balb/c mice prior to *A. baumannii* pulmonary infection resulted in an acute lethal respiratory disease that was associated with high *A. baumannii* burden and systemic dissemination (van Faassen et al., 2007). Nevertheless, the expression of Gr-1 on non-neutrophils has raised concern regarding the use of RB6-8C5 to induce neutropenia, as the results of studies that used this antibody may be confounded by the unintended depletion of other Gr-l-expressing cells. On the other hand, the mAb 1A8 binds specifically to Ly-6G^+^ cells (Fleming et al., 1993), and its administration has no impact on Gr-1-expressing cells (Daley et al., 2008).

The importance of neutrophils in the response to *A. baumannii* invasion in dermal tissue is not well established. Neutrophils begin arriving at a tissue injury site within minutes, in association with trauma or disruption in the integrity of the skin barrier. These phagocytic cells orchestrate other cells of the innate immune system, circulating through the body and extravasating to sites of infection and injury where they perform important roles in host defense. We showed that mAb 1A8 reduces the number of circulating neutrophils in the blood of Balb/c mice using differential leukocyte counts and flow cytometry. Likewise, IHC, neutrophil counts, and MPO levels revealed that this antibody decreases neutrophil infiltration into wounded tissue. Our results indicate that neutrophil deficiency exacerbates *A. baumannii* infection. The reported short lifespan of these cells, together with their potent antimicrobial functions, have limited our understanding of their role in immunity to that of effector cells. However, neutrophils also control adaptive immune responses during acute and chronic microbial infections (Sporri et al., 2008; Mantovani et al., 2011). For instance, neutrophils play an important role regulating natural killer (NK) cell maturation (Jaeger et al., 2012). NK cells are closely related to neutrophils, playing a crucial role in host defense against *A. baumannii* pulmonary infection (Tsuchiya et al., 2012). Also, neutrophils are critical activators of NK cells in mice, acting against *Legionella pneumophila* infection (Sporri et al., 2008).

We observed that 1A8-treated mice infected with *A. baumannii* are subject to a higher bacterial burden than controls. Histological analysis demonstrated that the presence of high numbers of bacteria in 1A8-treated animals was due to a reduction in neutrophil recruitment. Neutrophils are potent effectors of the innate immune response and contribute to protection in bacterial infections, including *A. baumannii* pneumonia (van Faassen et al., 2007), through their direct antimicrobial capacity and the production of cytokines and chemokines that instruct the recruitment and activation of other immune cells (Mantovani et al., 2011). We observed a significant early decrease in the levels of IFN-γ in the cutaneous tissue of 1A8-treated animals infected with *A. baumannii*; this cytokine produced by NK cells is an important activator of macrophages and inducer of MHC class II molecule expression which are important to control infections caused by extracellular microbes. Perhaps, the reduced production of IFN-γ in neutropenic animals impairs the effector functions of macrophages including phagocytosis, reactive oxygen species synthesis, bacterial antigen processing, and presentation to T cells interfering with the amplification of the immune response resulting in delayed resolution of the bacteria from wounded tissue.

TNF-α, a cytokine mostly produced by macrophages, was found to be similarly secreted in control and neutropenic mice. However, it is well-known that IL-1β regulates IL-6 production in human monocytes (Tosato and Jones, 1990). Thus, high levels of IL-1β and IL-6 suggest that macrophages supplemented the low number of neutrophils being unable to successfully eliminate *A. baumannii* from skin tissue. This is a plausible scenario because macrophages are important in early host defense against *A. baumannii* infection through the efficient phagocytosis and killing of the bacterium limiting its replication (Qiu et al., 2012; Bruhn et al., 2015). A previous study in our laboratory showed an increase in macrophage recruitment to the lungs in mice treated with cyclophosphamide, suggesting that the massive infiltration of these phagocytic cells may compensate for the reduction of neutrophils early during infection (Manepalli et al., 2013). In this regard, macrophages express diverse pathogen pattern recognition molecules (e.g., Toll-like receptors) to identify and engulf microbes and their derivatives (Steele et al., 2003; Qiu et al., 2012) being capable of clearing a low inoculum of microbes without the recruitment of neutrophils (Marriott and Dockrell, 2007). In addition, macrophages modulate microbial invasion by releasing pro-inflammatory cytokines and depend on the action of neutrophils to eliminate *A. baumannii*. This is also evinced by the reduced production of superoxide, part of the antimicrobial armamentarium of neutrophils. Also, *A. baumannii* possess a superoxide dismutase that confers resistance against the toxic effects of reactive oxygen species by effectively catalyzing the conversion of superoxide radicals (• O_2^-^_) into hydrogen peroxide and oxygen (Smith et al., 2007; Heindorf et al., 2014).

Previous studies of neutrophil function support both a positive (Devalaraja et al., 2000) and a negative (Ashcroft et al., 2000) role for these innate immune cells in wound healing. We observed that neutropenia does not affect tissue repair in uninfected animals but promotes late collagen expression and deposition in injured tissue. However, during *A. baumannii* infection, neutrophil-depletion delays wound healing since mAb 1A8-treated C57BL/6 mice exhibited slower tissue repair than control IgG2a-treated animals which could be possibly explained by longer persistence of the bacteria in the wounds of neutropenic animals. Surprisingly, neutropenic animals displayed high collagen expression and content in wounded cutaneous tissues. Neutrophils have been shown to produce collagenases that degrade collagen in an early wound, so a lack of neutrophils may directly result in increased ultimate quantitative collagen content of wounds (Steed, 1997). Another intriguing possibility is that an increase in macrophage recruitment and function, favorable to healing, in tandem with the depletion of neutrophils, accounts for the increased wound collagen content observed in neutrophil-depleted mice. Macrophages have proven to be key regulators of wound healing, with differential effects at various stages of wound healing (Koh and DiPietro, 2011).

## Conclusion

This is the first report that experimentally and specifically studies the impact of neutrophils in controlling *A. baumannii* cutaneous infection. MAb 1A8-induced neutropenia intensifies *A. baumannii*-skin invasion and disease progression *in vivo*. The depletion of neutrophils contributes to the higher bacterial burden found in murine cutaneous lesions during *A. baumannii* infection as well as delayed wound healing. Therefore, we hope that this study can aid in clarifying the role of neutrophils in regulating skin and soft tissue infections caused by *A. baumannii*.

## Conflict of Interest Statement

The authors declare that the research was conducted in the absence of any commercial or financial relationships that could be construed as a potential conflict of interest.
